# Uric acid to high-density lipoprotein cholesterol ratio as a novel biomarker for sarcopenia: a national study with machine learning insights

**DOI:** 10.1093/gerona/glag132

**Published:** 2026-05-22

**Authors:** Ruonan Yang, Zesong Cheng, Pei Yang, Xiujuan Yang, Wanlin Liao, Siwei Zhai

**Affiliations:** Department of Medical Quality Management, Chengdu Seventh People’s Hospital, Chengdu, China; Institute of Rare Diseases, West China Hospital, Sichuan University, Chengdu, China; Department of Endocrinology, The First Affiliated Hospital of University of South China, Hengyang, China; Department of Medical Quality Management, Chengdu Seventh People’s Hospital, Chengdu, China; Department of Medical Quality Management, Chengdu Seventh People’s Hospital, Chengdu, China; Department of Medical Quality Management, Chengdu Seventh People’s Hospital, Chengdu, China; (Medical Sciences Section)

**Keywords:** Sarcopenia, Uric acid, High-density lipoprotein cholesterol, Biomarker, Machine learning

## Abstract

**Background:**

Sarcopenia is a major age-related health burden. Although the uric acid to high-density lipoprotein cholesterol ratio (UHR) has been linked to sarcopenia, its underlying pathways and clinical utility remain unclear.

**Methods:**

We analyzed 9853 adults from the China Health and Retirement Longitudinal Study. Sarcopenia was defined by Asian Working Group for Sarcopenia 2019 Consensus criteria. We assessed the UHR-sarcopenia relationship using multivariable logistic regression and restricted cubic splines. Mediation analyses examined roles of insulin resistance (TyG index) and renal function (eGFR). Five machine learning algorithms were developed and evaluated in training (70%) and testing (30%) sets. Feature selection was performed using Boruta and LASSO, and model interpretability was assessed using SHapley Additive exPlanations (SHAP).

**Results:**

Higher UHR was independently associated with lower odds of sarcopenia, with an adjusted odds ratio per-standard deviation increase of 0.75 (95% CI: 0.69–0.81; p < .001), and showed a nonlinear dose-response relationship. UHR correlated with better handgrip strength and chair-stand performance. The TyG index and eGFR mediated 16.44% and 2.03% of this association, respectively. In machine learning analyses, ROC-AUC values ranged from 0.73 to 0.77, with XGBoost showing the best performance. SHAP analysis based on the XGBoost model further identified UHR as the second most important predictor of sarcopenia, following age.

**Conclusions:**

Higher UHR was associated with lower sarcopenia risk in Chinese middle-aged and older adults, partly through metabolic and renal pathways. UHR may serve as a simple biomarker for sarcopenia risk stratification.

## Introduction

The accelerating pace of global population aging has placed age-related musculoskeletal disorders at the forefront of public health challenges.^1^ Sarcopenia, a syndrome characterized by the progressive and generalized loss of skeletal muscle mass, strength, and function, imposes a substantial burden on individuals and healthcare systems.^2–4^ It is a primary driver of frailty, physical disability, loss of independence, and is strongly associated with increased risks of falls, fractures, hospitalization, and mortality.^5^^,^^6^ The economic and societal costs linked to sarcopenia and its complications underscore an urgent need for effective strategies for early identification and prevention. However, current diagnostic criteria often rely on tools such as dual-energy X-ray absorptiometry (DXA) and comprehensive physical performance batteries, which are resource-intensive and not readily scalable for widespread community or primary care screening.^7^^,^^8^ This challenge thus underscores a critical demand for accessible, cost-effective, and integrative biomarkers that can flag individuals at elevated risk, enabling timely and targeted interventions.

In the search for such biomarkers, metabolic dysregulation has emerged as a pivotal pathway in the pathogenesis of sarcopenia. Circulating uric acid (UA) and high-density lipoprotein cholesterol (HDL-C) represent 2 key metabolic markers with opposing biological actions relevant to muscle homeostasis.^9^^,^^10^ The UA to HDL-C ratio (UHR) integrates these counterbalancing signals into a single composite metric, theoretically offering a superior gauge of the systemic pro-oxidant/antioxidant milieu. This integrative capacity has established UHR as a robust predictor across various cardiometabolic conditions, including insulin resistance, non-alcoholic fatty liver disease, and cardiovascular events.^11^^,^^12^

Critically, the relevance of UHR to musculoskeletal aging is now gaining empirical support. A recent longitudinal study by Li et al.^13^ utilizing the China Health and Retirement Longitudinal Study (CHARLS) cohort confirmed a significant inverse association between higher UHR levels and a lower risk of incident sarcopenia over a 4-year follow-up period. This finding provides valuable epidemiological evidence linking UHR to sarcopenia development. However, it also highlights the pressing need to elucidate the mechanistic pathways involved and to evaluate its predictive value within a multidimensional clinical context—key aspects that remain unclear and currently impede its full biological understanding and clinical deployment.

First, the mechanistic pathways underlying the UHR-sarcopenia relationship remain largely unquantified—a “black box” within an otherwise significant correlation. Insulin resistance and declining renal function are established, intertwined risk factors for muscle loss and are intimately linked to both UA and HDL-C metabolism.^11^^,^^14^^,^^15^ The extent to which the triglyceride-glucose (TyG) index, a validated surrogate for insulin resistance, and estimated glomerular filtration rate (eGFR) mediate the protective effect of UHR is unknown. Formal causal mediation analysis is required to test these plausible biological pathways and assess their contribution, a step essential for establishing biological plausibility beyond statistical association.

Second, and of direct relevance to clinical application, the predictive utility and relative importance of UHR within a multidimensional clinical profile are undefined. Traditional regression models, while confirming an independent association, cannot adjudicate the rank-order importance of UHR against a comprehensive set of readily available clinical and laboratory variables. Determining whether UHR provides incremental value for risk stratification is a prerequisite for considering its adoption in screening algorithms. Advanced, interpretable ML approaches are uniquely suited to address this question by performing rigorous feature selection and quantifying variable importance within complex, nonlinear interactions.^16^

Therefore, to address these unresolved questions and advance the understanding of UHR’s role, the present study was designed with 3 specific aims: (1) To evaluate the independent, dose-response association between UHR and sarcopenia in a large, nationally representative sample of middle-aged and older Chinese adults. (2) To quantify the proportion of this association mediated by insulin resistance (via the TyG index) and renal function (via eGFR) using formal causal mediation analysis. (3) To determine the relative importance of UHR for sarcopenia prediction within a broad set of clinical covariates by employing an interpretable ML pipeline, thereby evaluating its potential utility as a component of a future risk assessment tool.

## Methods

### Study design and population

This cross-sectional study utilized data from the 2015 wave of the CHARLS, a nationally representative survey of community-dwelling adults aged ≥45 years in China.^17^ We excluded participants who lacked fasting blood test results, had incomplete data for sarcopenia assessment, or had missing information on key covariates (Figure S1). The final analytical sample comprised 9853 participants. All analyses incorporated CHARLS sampling weights, stratification, and clustering variables to account for the complex survey design and produce nationally representative estimates.^17^

### Assessment of the exposure: uric acid to HDL-C ratio

The primary exposure was the UHR. After an overnight fast, venous blood samples were collected by trained medical staff from the Chinese Center for Disease Control and Prevention (China CDC) following a standardized protocol. Serum UA concentration was measured in mg/dL using the UA Plus method, and HDL-C concentration was quantified in mg/dL via an enzymatic colorimetric assay at a central laboratory. UHR was calculated as UA (mg/dL) divided by HDL-C (mg/dL). For regression analyses, UHR was examined as a continuous variable standardized to Z-scores and categorized into quartiles.

### Assessment of the outcome: sarcopenia and continuous muscle parameters

Sarcopenia was defined according to the 2019 Asian Working Group for Sarcopenia (AWGS 2019) consensus. Muscle strength was assessed by handgrip strength (kg) using a dynamometer, with low muscle strength defined as <28 kg for men and <18 kg for women. Appendicular skeletal muscle mass (ASM, kg) was estimated using a validated Chinese-specific anthropometric equation that has demonstrated strong agreement with DXA in multiple studies.^18^ The equation is as follows: ASM = 0.193 × weight (kg) + 0.107 × height (cm) − 4.157 × sex − 0.037 × age (years) − 2.631, where sex was coded as 1 for males and 2 for females. The appendicular skeletal muscle mass index (ASMI, kg/m^2^) was calculated as ASM divided by height squared. Low muscle mass was defined as ASMI < 7.0 kg/m^2^ for men and < 5.4 kg/m^2^ for women.^19^ Low physical performance was defined as gait speed <1.0 m/s or 5 chair-stand time ≥12 seconds. Participants were classified as having sarcopenia if they presented with low ASMI plus either low handgrip strength or low physical performance.^20^

To complement the primary dichotomous outcome and to examine associations with specific components of muscle health, we also analyzed handgrip strength (kg), gait speed (m/s), five-repetition chair-stand time (s), and appendicular skeletal muscle mass index (ASMI, kg/m^2^) as continuous secondary outcomes.

### Covariates

Covariates were selected a priori based on established associations with sarcopenia. These included demographic factors (age, sex, residence, education, and relationship status), lifestyle behaviors (smoking, alcohol consumption), physician-diagnosed comorbidities (hypertension, hyperlipidemia, cardiovascular disease), and laboratory parameters (hemoglobin, hematocrit, mean corpuscular volume, platelet count, blood urea nitrogen, low-density lipoprotein cholesterol [LDL-C], total cholesterol, cystatin C, and glycosylated hemoglobin (HbA1c)).

### Statistical analysis for epidemiological associations

All traditional statistical analyses accounted for the CHARLS complex survey design using appropriate weights. The association between UHR and sarcopenia was assessed using multivariable logistic regression across 3 sequentially adjusted models: Model 1 (unadjusted); Model 2 (adjusted for demographics, lifestyle, and comorbidities); Model 3 (fully adjusted, including laboratory parameters). Results are presented as ORs with 95% confidence intervals (CIs). To ensure the stability of the regression models, we assessed multicollinearity among all covariates by calculating the variance inflation factor (VIF). A VIF value ≥ 10 was considered indicative of problematic multicollinearity.^21^ In our fully adjusted model (Model 3), all VIF values were below 10, indicating no substantial multicollinearity among the covariates. Associations between continuous UHR and secondary continuous outcomes—specifically handgrip strength, gait speed, chair-stand time, and ASMI—were assessed using multivariable linear regression. A dose-response relationship between UHR and sarcopenia was examined using restricted cubic splines (RCS) with 4 knots [4]. To evaluate whether the association between UHR and sarcopenia varied across different population strata, we performed pre-specified subgroup analyses stratified by sex, age (< 60 vs ≥ 60 years), residence (urban vs rural), hypertension (yes/no), hyperlipidemia (yes/no), and pain status (pain-free, single-site, or multisite). Additionally, we assessed effect modification by including multiplicative interaction terms (UHR × each stratifying variable) in the logistic regression models, with statistical significance determined by the Wald test.

### Causal mediation analysis

To quantify mechanistic pathways, we performed formal causal mediation analysis within the counterfactual framework.^22^ The mediators were: the triglyceride-glucose (TyG) index [Ln(fasting triglycerides (mg/dL) × fasting glucose (mg/dL)/2)]^23^ for insulin resistance; and the estimated glomerular filtration rate (eGFR) calculated using the CKD-EPI creatinine equation^24^ for renal function. The total effect (TOT) of UHR on sarcopenia was decomposed into an average causal mediation effect (ACME) and an average direct effect (ADE), with the proportion mediated calculated as ACME/TOT. All models adjusted for covariates in the fully adjusted logistic model, with 95% CIs estimated using 5000 non-parametric bootstrap samples.

### Machine learning

We implemented a supervised ML pipeline to evaluate the predictive importance of UHR. All continuous features were Z-score standardized. The dataset was randomly split into a training set (70%) and a held-out test set (30%). Feature selection was conducted exclusively in the training set using 2 complementary and independent approaches. The Boruta algorithm, a random forest (RF) based method, was applied as a sensitivity analysis to identify all features potentially relevant to sarcopenia by comparing original variables with randomized shadow features. In parallel, LASSO regression with 10-fold cross-validation (optimal λ selected by minimizing binomial deviance) was used to select a parsimonious set of predictors optimized for predictive performance.

The final set of variables for model development was determined based on LASSO results, given the primary aim of optimizing predictive accuracy and model sparsity, while Boruta was used solely to provide complementary evidence on feature relevance.

Five ML algorithms—logistic regression (LR), decision tree (DT), RF, support vector machine (SVM), and extreme gradient boosting (XGBoost)—were trained and tuned using 5-fold cross-validated grid search in the training set. Model performance was evaluated in the held-out test set using multiple metrics, including the area under the receiver operating characteristic curve (ROC-AUC), precision-recall AUC (PR-AUC), accuracy, balanced accuracy, sensitivity, specificity, positive predictive value (PPV), negative predictive value (NPV), F1-score (F-meas), Matthews correlation coefficient (MCC), Youden’s index (J-index), and Cohen’s kappa (kap). The models were compared based on ROC-AUC in the test set, and the model with the highest ROC-AUC was selected as the best-performing model. Model interpretability and feature importance were derived using SHapley Additive exPlanations (SHAP) values.^25^

## Results

### Baseline characteristics

The baseline characteristics of the 9853 participants, stratified by UHR quartiles, are presented in Table 1. Participant age and the proportion of males increased progressively across higher UHR quartiles (both *p* < .001). Concurrently, the prevalence of hypertension and hyperlipidemia rose with higher UHR, whereas the prevalence of sarcopenia declined from 47.7% in the lowest quartile (Q1) to 36.2% in the highest quartile (Q4) (*p* < .001).

**Table 1:** Baseline characteristics of participants included in this study.

	Q1	Q2	Q3	Q4	*p*
*N*	2547	2464	2442	2400	
Sex = male (%)	608 (23.9)	915 (37.1)	1301 (53.3)	1680 (70.0)	<.001
Age (mean (SD))	59.54 (9.99)	59.96 (9.87)	60.75 (9.62)	61.53 (10.05)	<.001
Residence = rural (%)	2116 (83.1)	1912 (77.6)	1810 (74.1)	1657 (69.0)	<.001
**Relationship status (%)**					.005
Divorced	12 (0.5)	25 (1.0)	30 (1.2)	20 (0.8)	
Married	2179 (85.6)	2129 (86.4)	2132 (87.3)	2122 (88.4)	
Never	18 (0.7)	16 (0.6)	20 (0.8)	17 (0.7)	
Widowed	338 (13.3)	294 (11.9)	260 (10.6)	241 (10.0)	
Smoke = yes (%)	88 (3.5)	125 (5.1)	146 (6.0)	219 (9.1)	<.001
**Alcohol (%)**					<.001
Infrequent	221 (8.7)	209 (8.5)	210 (8.6)	214 (8.9)	
No	1818 (71.4)	1678 (68.1)	1487 (60.9)	1422 (59.2)	
Ofen	508 (19.9)	577 (23.4)	745 (30.5)	764 (31.8)	
**Job (%)**					<.001
Agricultural	865 (34.0)	788 (32.0)	677 (27.7)	575 (24.0)	
Both	126 (4.9)	135 (5.5)	153 (6.3)	162 (6.8)	
No	1435 (56.3)	1376 (55.8)	1401 (57.4)	1426 (59.4)	
Non-agricultural	121 (4.8)	165 (6.7)	211 (8.6)	237 (9.9)	
Hypertension = yes (%)	409 (16.1)	482 (19.6)	602 (24.7)	762 (31.8)	<.001
Hyperlipidemia = yes (%)	184 (7.2)	190 (7.7)	276 (11.3)	378 (15.8)	<.001
Cardiovascular disease = yes (%)	279 (11.0)	265 (10.8)	276 (11.3)	337 (14.0)	.001
**Pain (%)**					<.001
Pain-free	1671 (65.6)	1702 (69.1)	1725 (70.6)	1760 (73.3)	
Single-site	117 (4.6)	114 (4.6)	121 (5.0)	93 (3.9)	
Multisite	759 (29.8)	648 (26.3)	596 (24.4)	547 (22.8)	
Sarcopenia = yes (%)	1214 (47.7)	968 (39.3)	901 (36.9)	870 (36.2)	<.001
White blood cell (in thousands)	5.58 (1.78)	5.79 (1.68)	6.05 (1.75)	6.37 (1.93)	<.001
Hemoglobin (g/dL)	13.22 (1.75)	13.60 (1.87)	13.97 (2.02)	14.22 (1.96)	<.001
Hematocrit (%)	40.14 (5.25)	41.22 (5.43)	42.17 (5.76)	42.79 (5.76)	<.001
Mean corpuscular volume (fl)	91.26 (7.76)	91.35 (7.52)	91.62 (7.32)	91.41 (8.07)	.404
Platelet count (10^9^/L)	207.00 (75.36)	204.49 (75.50)	207.12 (73.86)	201.25 (75.62)	.020
Triglycerides (mg/dL)	101.62 (52.03)	125.78 (70.77)	145.35 (86.82)	182.21 (110.91)	<.001
Creatinine (mg/dL)	0.71 (0.19)	0.76 (0.20)	0.82 (0.32)	0.92 (0.39)	<.001
Blood urea nitrogen (mg/dL)	14.96 (4.45)	15.12 (4.29)	15.41 (4.74)	16.07 (5.23)	<.001
HDL cholesterol (mg/dL)	61.24 (12.05)	53.50 (8.88)	48.48 (8.00)	41.94 (7.20)	<.001
LDL cholesterol (mg/dL)	104.54 (28.70)	105.89 (29.00)	102.68 (28.24)	97.75 (29.86)	<.001
Total cholesterol (mg/dL)	189.82 (35.22)	187.09 (36.16)	182.36 (35.95)	177.68 (38.34)	<.001
Glucose (mg/dL)	96.60 (28.63)	99.66 (29.74)	102.12 (32.39)	103.67 (29.13)	<.001
Uric acid (mg/dL)	3.59 (0.74)	4.49 (0.75)	5.21 (0.86)	6.46 (1.25)	<.001
Cystatin C (mg/L)	0.76 (0.18)	0.81 (0.17)	0.87 (0.22)	0.97 (0.31)	<.001
Glycated hemoglobin (%)	5.91 (1.01)	5.96 (0.94)	6.02 (1.06)	6.07 (0.99)	<.001

Abbreviations: HDL, high-density lipoprotein; LDL, low-density lipoprotein.

### Association between UHR and sarcopenia

**Figure 1:**
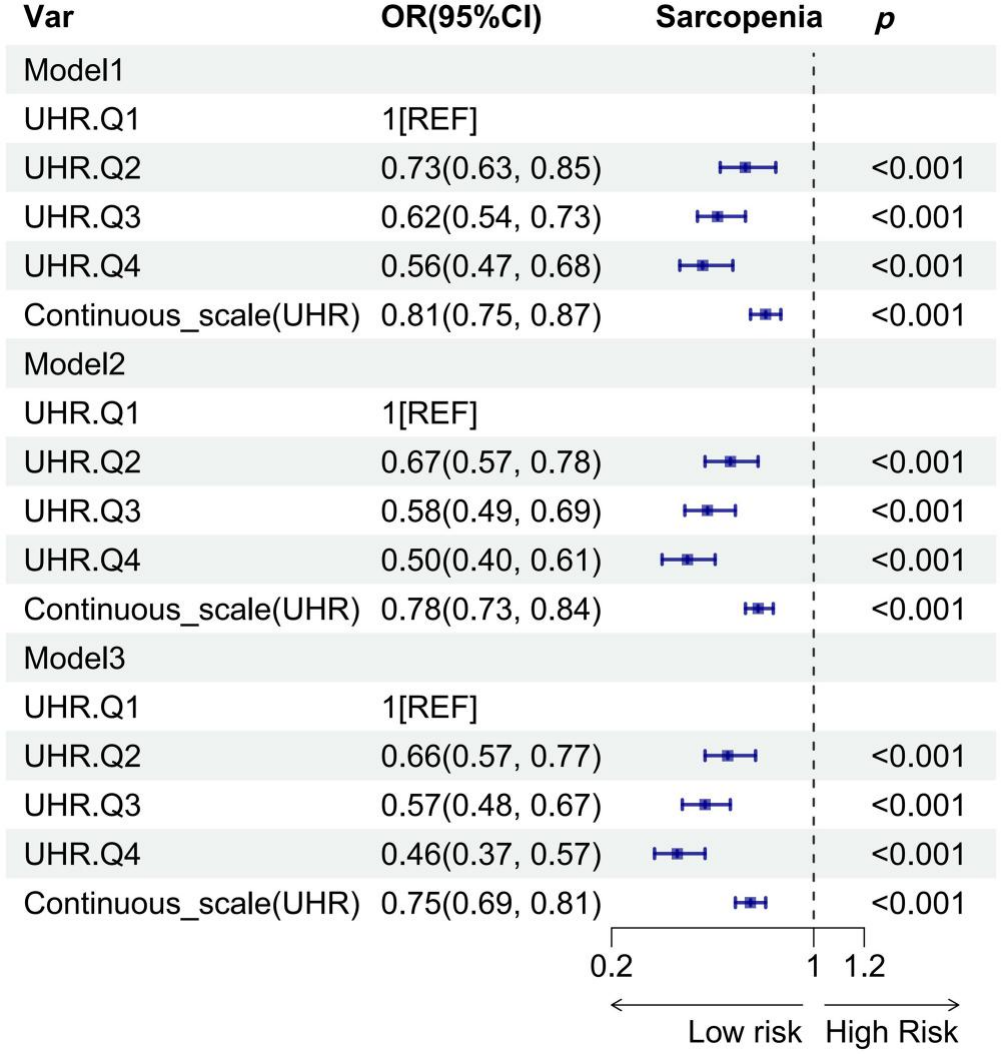
Effects of UHR on sarcopenia. Forest plot showing the association between uric acid to HDL-C ratio (UHR) and sarcopenia across 3 sequentially adjusted models. UHR was analyzed both as quartiles (Q1-Q4, with Q1 as reference) and as a continuous variable (per standard deviation increase). Model 1 was unadjusted; Model 2 was adjusted for demographics, lifestyle factors, and comorbidities; Model 3 was fully adjusted including all laboratory parameters. Odds ratios (ORs) and 95% confidence intervals (CIs) are displayed. All *p* values for trend across quartiles and for continuous UHR were <.001 in the fully adjusted model.

**Figure 2:**
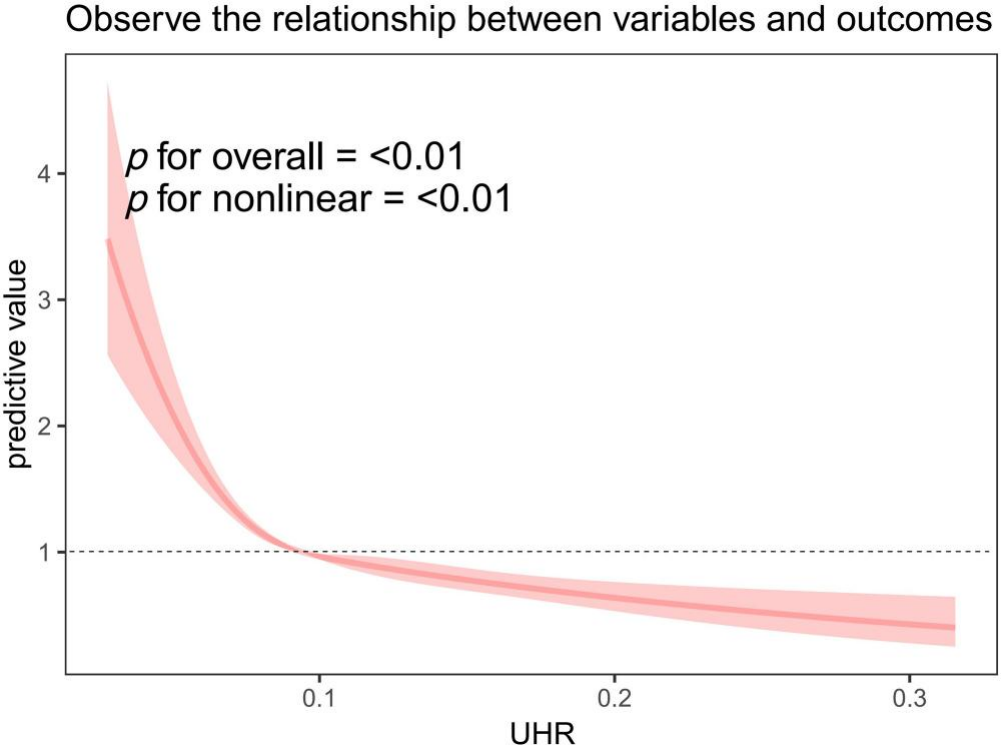
Dose-effect relationship between UHR and sarcopenia. Restricted cubic spline analysis illustrating the dose‑response association between uric acid to HDL-C ratio (UHR) and sarcopenia. The solid line represents the adjusted odds ratio (OR), and the shaded area indicates the 95% confidence interval. The reference point was set at the median UHR value. The analysis was adjusted for all covariates included in the fully adjusted model (Model 3). Both the overall association (*p* for overall < .01) and the nonlinear component (*p* for nonlinear < .01) were statistically significant, indicating a nonlinear inverse relationship.

In weighted analyses accounting for the complex survey design, a higher UHR was independently associated with lower odds of sarcopenia. In the fully adjusted model (Model 3), each standard deviation increase in UHR corresponded to an OR of 0.75 (95% CI: 0.69-0.81; *p* < .001, Figure 1). A graded, inverse association was evident across sex‑specific UHR quartiles (*p* for trend < .001). RCS analysis confirmed a significant nonlinear dose‑response relationship (*p*‑nonlinear < .01; Figure 2).

### Associations between UHR and continuous muscle health parameters

To further elucidate the relationship between UHR and specific domains of muscle health, we examined its association with key continuous measures: handgrip strength (muscle strength), five-repetition chair-stand time (muscle function), gait speed (physical performance), and appendicular skeletal muscle mass index (ASMI, muscle mass). The fully adjusted linear regression results are summarized in Table 2.

**Table 2:** Association between the uric acid to HDL-C ratio (UHR) and continuous measures of muscle health.

Variables	Handgrip strength		Gait speed		Chair-stand time		ASMI	
	β (95% CI)	*p*	β (95% CI)	*p*	β (95% CI)	*p*	β (95% CI)	*p*
UHR Q1	Ref		Ref		Ref		Ref	
UHR.Q2	1.70 (0.28, 3.11)	.019	4.61 (−4.29, 13.51)	.309	−0.05 (−0.31, 0.22)	.736	−1.91 (−6.18, 2.35)	.378
UHR.Q3	4.32 (0.30, 8.33)	.035	1.04 (−0.37, 2.45)	.147	−0.34 (−0.60, −0.08)	.010	−12.90 (−39.10,13.30)	.334
UHR.Q4	3.19 (1.63, 4.76)	>.001	−0.60 (−1.89, 0.68)	.358	−0.22 (−0.52, 0.08)	.154	−4.00 (−13.10, 5.10)	.388
Continuous_scale (UHR)	0.88 (0.21, 1.54)	.010	−0.45 (−1.03, 0.13)	.13	−0.03 (−0.14, 0.08)	.596	−1.71 (−5.58, 2.15)	.383

Abbreviations: ASMI, appendicular skeletal muscle mass index; CI, confidence interval; Q, quartile; UHR, uric acid to high-density lipoprotein cholesterol ratio.

A higher UHR demonstrated a robust and significant association with greater handgrip strength. Compared to the lowest quartile (Q1), participants in Q2, Q3, and Q4 showed progressively higher grip strength (eg, Q4: β  =  3.19 kg, 95% CI: 1.63 to 4.76; *p* < .001). When analyzed as a continuous variable, each standard deviation increase in UHR was associated with a 0.88 kg (95% CI: 0.21 to 1.54; *p *= .01) increase in handgrip strength.

Regarding muscle function, a higher UHR (Q3) was significantly associated with a shorter time to complete 5 chair-stands (β = −0.34 s, 95% CI: −0.60 to −0.08; *p *= .01), indicating better lower-body functional performance. However, no statistically significant associations were observed between UHR and gait speed or ASMI in the fully adjusted models (all *p *> .05).

### Machine learning for feature importance

Given the cross-sectional nature of our data, the primary aim of applying ML was not to develop a prognostic prediction model but to evaluate the relative importance of UHR within a comprehensive set of clinical and laboratory variables for identifying prevalent sarcopenia status. Feature selection was performed using 2 complementary approaches. First, the Boruta algorithm was conducted as a sensitivity analysis to identify all features potentially relevant to sarcopenia (Figure S2). Boruta excluded platelet count and LDL cholesterol from consideration. Subsequently, LASSO regression was applied to select non-redundant predictors optimized for predictive performance, with the optimal λ determined by minimizing the binomial deviance (Figures S3 and S4). Because our primary aim was to optimize predictive performance, we prioritized LASSO results for model development. The final set of 9 variables selected by LASSO comprised: age, UHR, hematocrit, LDL cholesterol, cystatin C, mean corpuscular volume, HbA1c, platelet count, and blood urea nitrogen. This combined approach balances comprehensive feature exploration with predictive parsimony.

Model performance was evaluated on the held-out test set (30% of the sample). As shown in Figures S5-S7, all 5 algorithms achieved comparable discriminative ability, with ROC-AUC values ranging from 0.73 to 0.77. XGBoost yielded the highest ROC-AUC (0.77), followed by RF (0.76), LR (0.75), SVM (0.74), and DT (0.73). The corresponding accuracy, sensitivity, and specificity for XGBoost were 0.70, 0.70, and 0.71, respectively. The multi-metric performance heatmap (Figure S7) further illustrated the consistency of these findings across additional metrics, including precision, F1-score, and MCC, with XGBoost demonstrating superior or comparable performance across all evaluated metrics.

Model interpretability was assessed using both model-based feature importance and SHAP analysis within the XGBoost framework. Notably, UHR was consistently identified as the second most important predictor after age in both feature importance and SHAP-based interpretations.

The feature importance ranking derived from the built-in gain metric of XGBoost (Figure S8) identified the variables in the following order of importance: age, UHR, hematocrit, LDL cholesterol, cystatin C, mean corpuscular volume, HbA1c, platelet count, and blood urea nitrogen.

SHAP analysis (Figure S9) quantified the magnitude and direction of each feature’s contribution to model output, with higher UHR values associated with lower probability of sarcopenia according to the model. The SHAP summary plot indicated a similar ranking of variables as the gain-based feature importance, with age and UHR as the top 2 contributors, followed by hematocrit, cystatin C, LDL cholesterol, mean corpuscular volume, HbA1c, blood urea nitrogen, and platelet count.

Additionally, the SHAP force plot (Figure S10) illustrated individualized prediction explanations by showing how positive and negative contributions from each variable combined to generate the final model prediction.

These analyses collectively demonstrate that UHR provides substantial independent predictive information for sarcopenia, supporting its relevance even alongside conventional clinical and laboratory measures.

### Mediation analysis of the UHR-sarcopenia association

**Figure 3:**
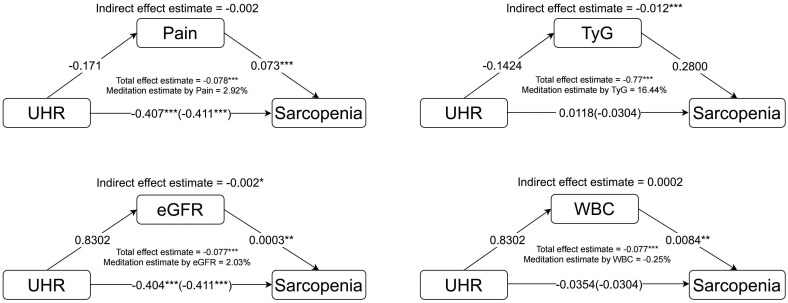
Mediating effects of number of pain sites, eGFR, TyG, and white blood cell count in the effects of UHR on sarcopenia. Causal mediation analysis decomposing the total effect of uric acid to HDL-C ratio (UHR) on sarcopenia into average direct effect (ADE) and average causal mediation effect (ACME) through 4 potential mediators: number of pain sites, estimated glomerular filtration rate (eGFR), triglyceride‑glucose (TyG) index, and white blood cell (WBC) count. Each mediator was tested in a separate model. The proportion mediated is shown as a percentage, with 95% confidence intervals estimated via 5000 non‑parametric bootstrap resamples. All models were adjusted for the full set of covariates used in the main analysis. Significant mediation was observed for TyG index (16.44%) and eGFR (2.03%), while pain sites and WBC count showed no significant mediating effects. *p < .05, **p < .01, ***p < .001.

We examined potential pathways underlying the UHR–sarcopenia association. Formal causal mediation analysis indicated that the triglyceride-glucose (TyG) index (insulin resistance) and estimated glomerular filtration rate (eGFR, renal function) accounted for 16.44% and 2.03% of the total effect, respectively (Figure 3). The mediation effects through systemic inflammation (white blood cell count) and pain status were not statistically significant.

### Subgroup analyses

Subgroup analyses were performed to examine whether the association between UHR and sarcopenia varied across different population strata. As shown in Table S1, the inverse association between higher UHR and lower odds of sarcopenia was consistently observed across all subgroups, including sex, age, residence, hypertension, hyperlipidemia, and pain status.

Specifically, for sex-stratified analysis, each standard deviation increase in UHR was associated with an OR of 0.74 (95% CI: 0.66-0.84) in females and 0.77 (95% CI: 0.69-0.86) in males (both *p* < .001). Formal interaction tests revealed no statistically significant effect modification by sex (*p*_interaction_ = .087), indicating that the protective effect of UHR against sarcopenia is consistent between men and women.

Similarly, no significant interactions were observed for age (*p*_interaction_ = .196), residence (*p*_interaction_ = .199), hypertension (*p*_interaction_ = .623), hyperlipidemia (*p*_interaction_ = .978), or pain status (*p*_interaction_ = .151). These findings suggest that the association between UHR and sarcopenia is robust and generalizable across key demographic and clinical subgroups.

## Discussion

This nationally representative study provides a comprehensive, multi-method investigation into the relationship between the UA to HDL-C ratio (UHR) and sarcopenia in middle-aged and older Chinese adults. Our principal findings are threefold. First, we confirm a significant, independent, and nonlinear inverse association between higher UHR levels and lower odds of sarcopenia. Second, formal mediation analysis suggests that insulin sensitivity (TyG index) and, to a lesser extent, renal function (eGFR) partially mediate this association. Third, and most notably, interpretable ML identified UHR as the second most important predictor of prevalent sarcopenia status after age, underscoring its substantial standalone value for risk stratification within a comprehensive clinical profile. Collectively, these findings position UHR as a robust, integrative biomarker linked to muscle health through plausible biological pathways.

The protective association we observed aligns with a nuanced understanding of the dual roles of UA and HDL-C in musculoskeletal aging. UA, often cited for its pro-oxidant effects, also serves as a major systemic antioxidant, scavenging reactive oxygen species and mitigating oxidative damage—a key driver of age-related muscle decline.^9^^,^^26^ Conversely, HDL-C exerts anti-inflammatory and endothelial-protective properties vital for maintaining muscle perfusion and metabolic homeostasis.^27^ The UHR metric likely captures a dynamic balance between these countervailing forces. Our results imply that a higher ratio, potentially reflecting a favorable antioxidant milieu or a specific metabolic state, is associated with better muscular outcomes. This perspective may help reconcile inconsistencies in prior literature where isolated UA levels have shown variable links to muscle mass,^28^ suggesting that a composite ratio offers a more holistic view of the metabolic environment pertinent to sarcopenia.

Critically, our analysis of continuous muscle parameters provides granular physiological support for this association. We found that a higher UHR was significantly correlated with greater handgrip strength and, in a specific quartile (Q3), with a shorter five-repetition chair-stand time. Handgrip strength is a core component of sarcopenia diagnosis and a powerful predictor of functional decline and mortality. The chair-stand test directly assesses lower-limb muscle power, endurance, and coordination. These findings strongly suggest that the protective effect of UHR extends beyond a reduced syndromic risk to directly supporting preserved muscle strength and physical function, which are paramount for autonomy and quality of life in older adults. A plausible mechanistic explanation is that the metabolic state reflected by UHR may help protect neuromuscular junction integrity, maintain myocellular membrane stability, or optimize energy substrate utilization, thereby translating into superior muscle performance.^29^ This offers a more direct and clinically relevant link than associations with dichotomous diagnostic outcomes alone.

To explore the pathways underlying this link, we employed causal mediation analysis. The finding that the TyG index (insulin resistance) mediated approximately 16% of UHR’s total effect provides a tangible metabolic explanatory route. Insulin resistance impairs muscle protein synthesis and promotes catabolism, constituting a central mechanism in muscle wasting.^30^^,^^31^ Thus, part of UHR’s apparent benefit may be attributable to its correlation with a more favorable systemic insulin sensitivity profile. The smaller yet significant mediation via eGFR further connects muscle health to overall metabolic clearance capacity,^32^ reinforcing the interplay between renal and musculoskeletal systems.

A key innovative aspect of our study is the objective evaluation of UHR’s relative importance using an interpretable ML framework. In a cross-sectional context, the primary utility of ML lies not in forecasting future events but in performing robust, data-driven feature selection and importance ranking within a high-dimensional set of variables. The SHAP analysis unequivocally demonstrated that UHR was the second most influential feature for discriminating current sarcopenia status, immediately after age. This finding carries immediate clinical implication: the simple, readily calculable UHR can yield incremental information about muscular health risk beyond the isolated interpretation of standard lipid or UA panels. It could serve as a pragmatic flag in routine geriatric assessment to identify individuals who may benefit from prioritized functional evaluation or targeted preventive strategies.

Notably, the association between UHR and sarcopenia was consistent across sexes, with no evidence of significant interaction, suggesting that this relationship is broadly generalizable to both male and female older adults.

Several limitations warrant consideration. First, the cross-sectional design precludes causal inference; longitudinal studies are needed to establish temporality. Second, UHR was measured at a single time point, not accounting for long-term fluctuations or the potential influence of medications affecting UA or lipid levels. Third, appendicular skeletal muscle mass was estimated using a validated anthropometric equation rather than a gold-standard imaging method such as DXA. This approach was necessitated by the CHARLS study design, as direct DXA measurements were not collected. Although this equation has demonstrated strong agreement with DXA in Chinese populations,^18^ measurement error is inevitable and may lead to non-differential misclassification of sarcopenia status, which would likely bias the association toward the null and potentially underestimate the true protective effect of UHR. Thus, although the overall conclusion of a protective association is supported, the observed effect size may be conservative. Fourth, our ML model, while robust internally, requires external validation in independent cohorts to confirm generalizability and calibrate performance. Finally, we could not differentiate between primary (age-related) and secondary sarcopenia, which may have distinct etiologies and metabolic profiles. Despite these constraints, the use of a large, nationally representative sample, extensive covariate adjustment, and a complementary analytical approach strengthens the internal validity of our conclusions.

In summary, this study elucidates the role of UHR as a novel, integrative biomarker associated with a lower risk of sarcopenia and better preservation of muscle strength and function in middle-aged and older adults. The association appears partly mediated through pathways involving insulin sensitivity and renal function. The prominent ranking of UHR in feature importance analysis highlights its potential utility for risk stratification. Future research should prioritize prospective cohorts to validate the longitudinal predictive value of UHR, experimental models to decipher its precise effects on neuromuscular biology, and external validation across diverse populations to assess generalizability. Incorporating UHR into geriatric assessment protocols could represent a simple, cost-effective step toward earlier identification of individuals at risk for functional decline, aligning with the imperative to develop actionable biomarkers for healthy aging.

## Conclusions

This study investigated the UA to HDL-C ratio (UHR) as a novel biomarker for sarcopenia in a national cohort of middle-aged and older adults. We confirmed an independent, inverse association between higher UHR and sarcopenia, partially mediated by insulin sensitivity and renal function. By employing interpretable ML, UHR was further validated as a key discriminating feature, second only to age, within a comprehensive clinical profile.

Our findings contribute to geriatric science by proposing UHR as an integrative metric that captures a metabolic balance relevant to muscle health, moving beyond isolated risk factors. From a clinical perspective, this easily calculable ratio shows promise for enhancing risk stratification in aging populations, potentially aiding in the early identification of individuals susceptible to functional decline.

The cross-sectional design precludes causal inference, and external validation of our model is needed. Future work should establish the longitudinal relationship between UHR and muscle loss, elucidate its underlying biological mechanisms, and test its utility in diverse populations and clinical settings.

This research underscores the value of UHR as a simple, informative tool that advances both the understanding and assessment of sarcopenia, supporting its potential integration into strategies for promoting musculoskeletal health in later life.

## Supplementary Material

glag132_Supplementary_Data

## Data Availability

Data are available in Peking University’s open research data service platform. Data for this study were sourced from the China Health and Retirement Longitudinal Study and are available at http://charls.pku.edu.cn/.
