# Supplementary material for: Uric acid to high-density lipoprotein cholesterol ratio as a novel biomarker for sarcopenia: a national study with machine learning insights
Source: J Gerontol A Biol Sci Med Sci. 2026 May 22;81(7):glag132. doi: 10.1093/gerona/glag132 (PMC13264432; doi:10.1093/gerona/glag132)
Supplement: glag132_Supplementary_Data [file glag132_supplementary_data.zip › Supplementary File.pdf]

Supplementary Material

Uric Acid to HDL-C Ratio as a Novel Biomarker for Sarcopenia: A National Study with Machine Learning Insights

Authors:

Ruonan Yang, MM1*#; Zesong Cheng, MM 2*; Pei Yang, MM 3; Xiujuan Yang, MM 1; Wanlin Liao, MM 1; Siwei Zhai, MM 1#;

* These authors contributed equally to this work.

# These authors are the corresponding authors.

Author affiliations:

1. Department of Medical Quality Management, Chengdu Seventh People's Hospital, Chengdu, Sichuan, China.

2. Institute of Rare Diseases, West China Hospital, Sichuan University, Chengdu, Sichuan, China.

3. Department of Endocrinology, The First Affiliated Hospital of University of South China, Hengyang, Hunan, China.

Corresponding author:

Siwei Zhai: Department of Medical Quality Management, Chengdu Seventh People's Hospital. Chengdu, Sichuan Province, China

E-mail: 11499022136@qq.com

Ruonan Yang: Department of Medical Quality Management, Chengdu Seventh People's Hospital. Chengdu, Sichuan Province, China

E-mail: 348629508@qq.com

Word count of main text: 4012 words

number of references: 32

number of data elements: A total of five figures and tables (2 tables and 3 figures)

eTable 1.Results of subgroup analyses based on sex, age, place of residence, hypertension, hyperlipidemia, and pain.

Var	UHR									
	Q1	Q2	*P*	Q3	*P*	Q4	*P*	Continuous UHR	*P*	$P_{interaction}$
Sex										
Female	1[Reference]	0.64(0.53-0.78)	<0.001	0.55(0.45-0.69)	<0.001	0.41(0.29-0.59)	<0.001	0.74(0.66-0.84)	<0.001	0.087
Male	1[Reference]	0.68(0.49-0.96)	0.027	0.58(0.43-0.80)	0.001	0.51(0.37-0.70)	<0.001	0.77(0.69-0.86)	<0.001	
Age										
＞60	1[Reference]	0.56(0.43-0.73)	<0.001	0.49(0.39-0.62)	<0.001	0.46(0.33-0.64)	<0.001	0.80(0.73-0.88)	<0.001	0.196
≤60	1[Reference]	0.75(0.58-0.98)	0.034	0.64(0.49-0.83)	0.001	0.40(0.29-0.55)	<0.001	0.66(0.58-0.74)	<0.001	
Residence										
Urban	1[Reference]	0.50(0.34-0.72)	<0.001	0.39(0.26-0.57)	<0.001	0.35(0.23-0.53)	<0.001	0.74(0.64-0.86)	<0.001	0.199
Rural	1[Reference]	0.73(0.62-0.86)	<0.001	0.65(0.54-0.78)	<0.001	0.50(0.40-0.63)	<0.001	0.76(0.69-0.82)	<0.001	
Hypertension										
Yes	1[Reference]	0.67(0.47-0.95)	0.027	0.66(0.46-0.94)	0.020	0.54(0.37-0.79)	0.001	0.79(0.68-0.90)	0.001	0.623
No	1[Reference]	0.65(0.54-0.78)	<0.001	0.53(0.44-0.64)	<0.001	0.42(0.32-0.54)	<0.001	0.73(0.67-0.79)	<0.001	
Hyperlipidemia										
Yes	1[Reference]	0.59(0.33-1.05)	0.072	0.60(0.36-0.99)	0.047	0.49(0.27-0.88)	0.017	0.74(0.59-0.94)	0.014	0.978
No	1[Reference]	0.66(0.56-0.78)	<0.001	0.56(0.47-0.68)	<0.001	0.46(0.37-0.57)	<0.001	0.75(0.69-0.81)	<0.001	
Pain										
Pain-free	1[Reference]	0.68(0.55-0.84)	<0.001	0.49(0.39-0.61)	<0.001	0.43(0.33-0.56)	<0.001	0.73(0.66-0.82)	<0.001	0.151
Single-site	1[Reference]	0.40(0.17-0.95)	0.039	0.44(0.18-1.07)	0.069	0.54(0.20-1.45)	0.221	0.71(0.48-1.05)	0.089	
Multisite	1[Reference]	0.66(0.49-0.89)	0.006	0.80(0.59-1.07)	0.137	0.60(0.39-0.93)	0.022	0.83(0.71-0.96)	0.013	

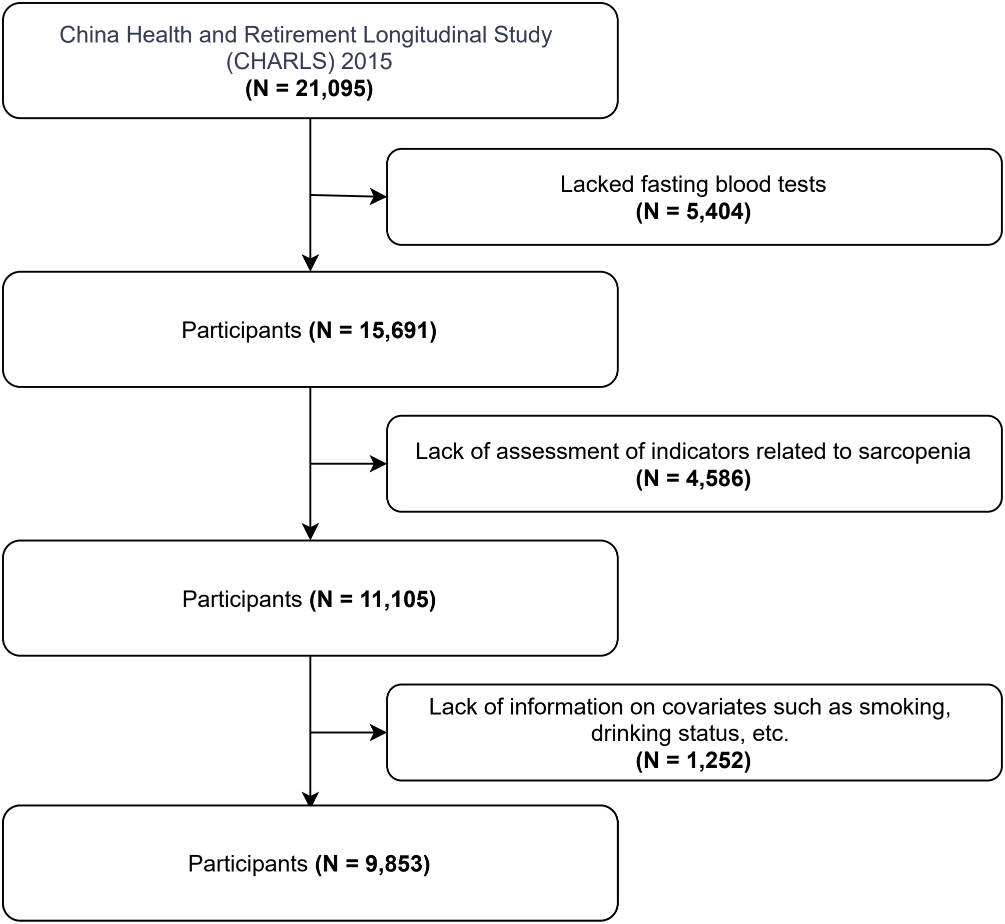

eFigure 1. Flowchart of sample selection for this study.

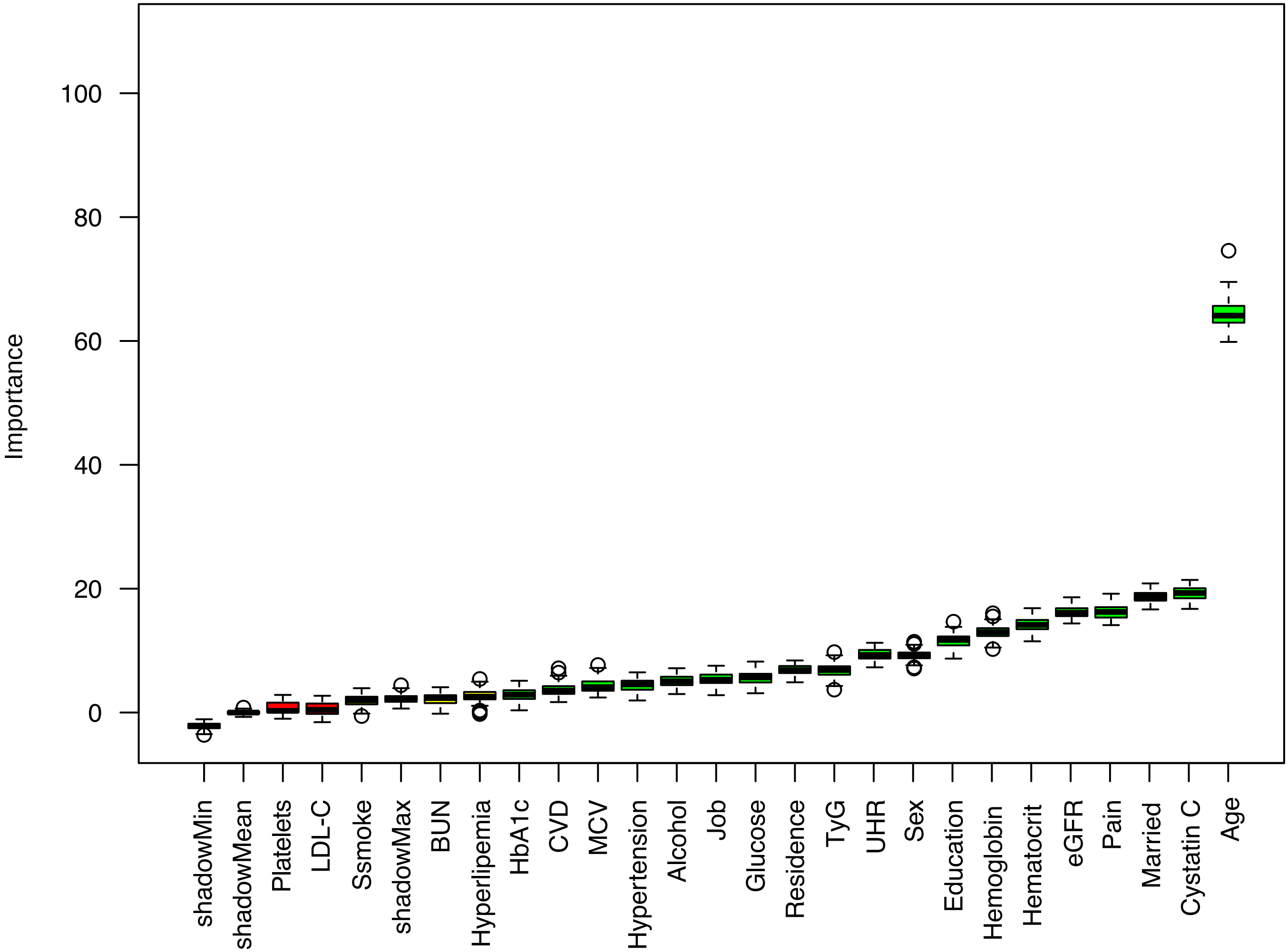

LDL-C: LDL Cholesterol
BUN: Blood Urea Nitrogen
HbA1c: Glycated Hemoglobin
CVD: Cardiovascular disease
MCV: Mean Corpuscular Volume

eFigure 2. Results of feature screening by Boruta analysis.

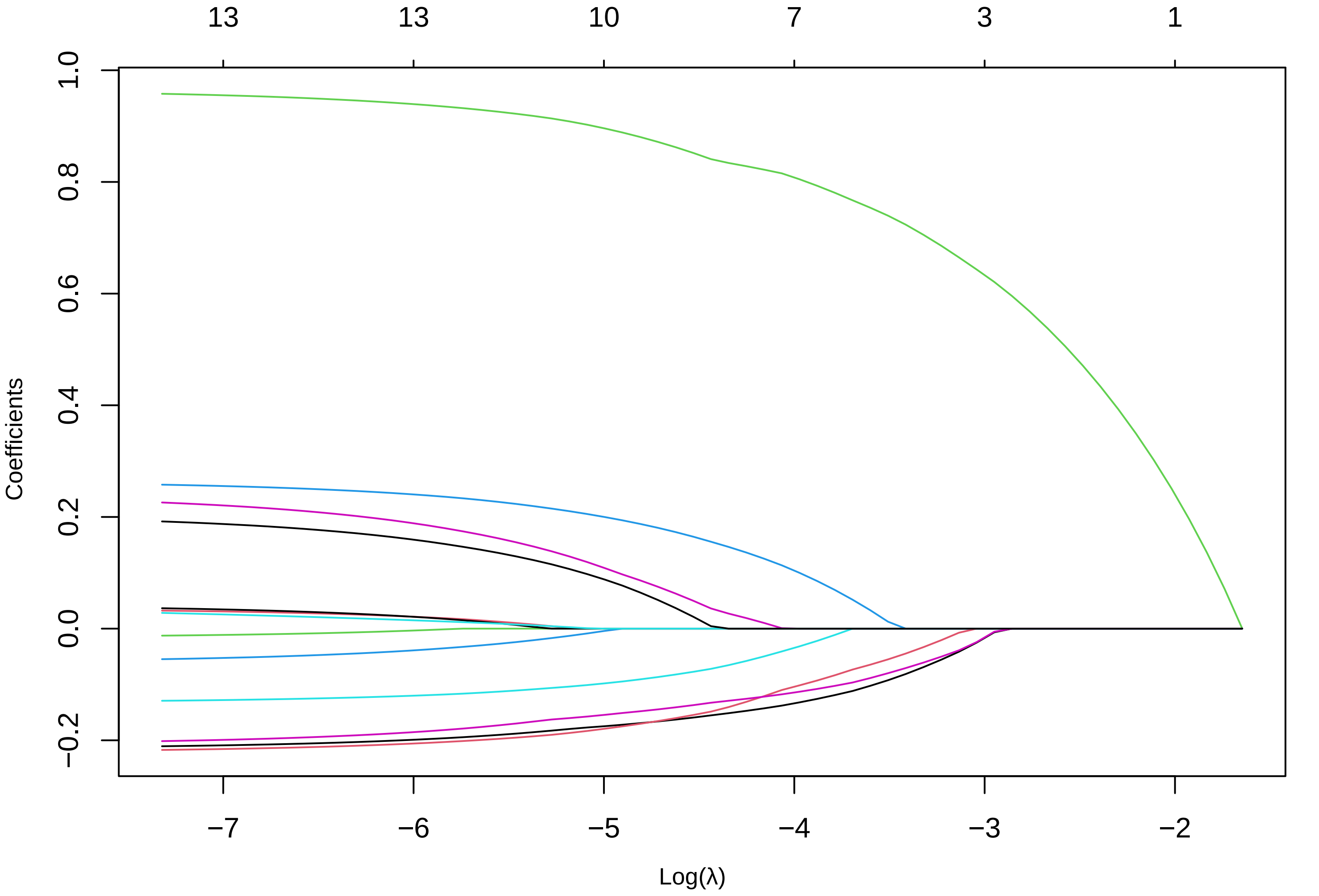

eFigure 3.Screening pathway diagram for sarcopenia risk factors (Lasso coefficient contraction trajectory).

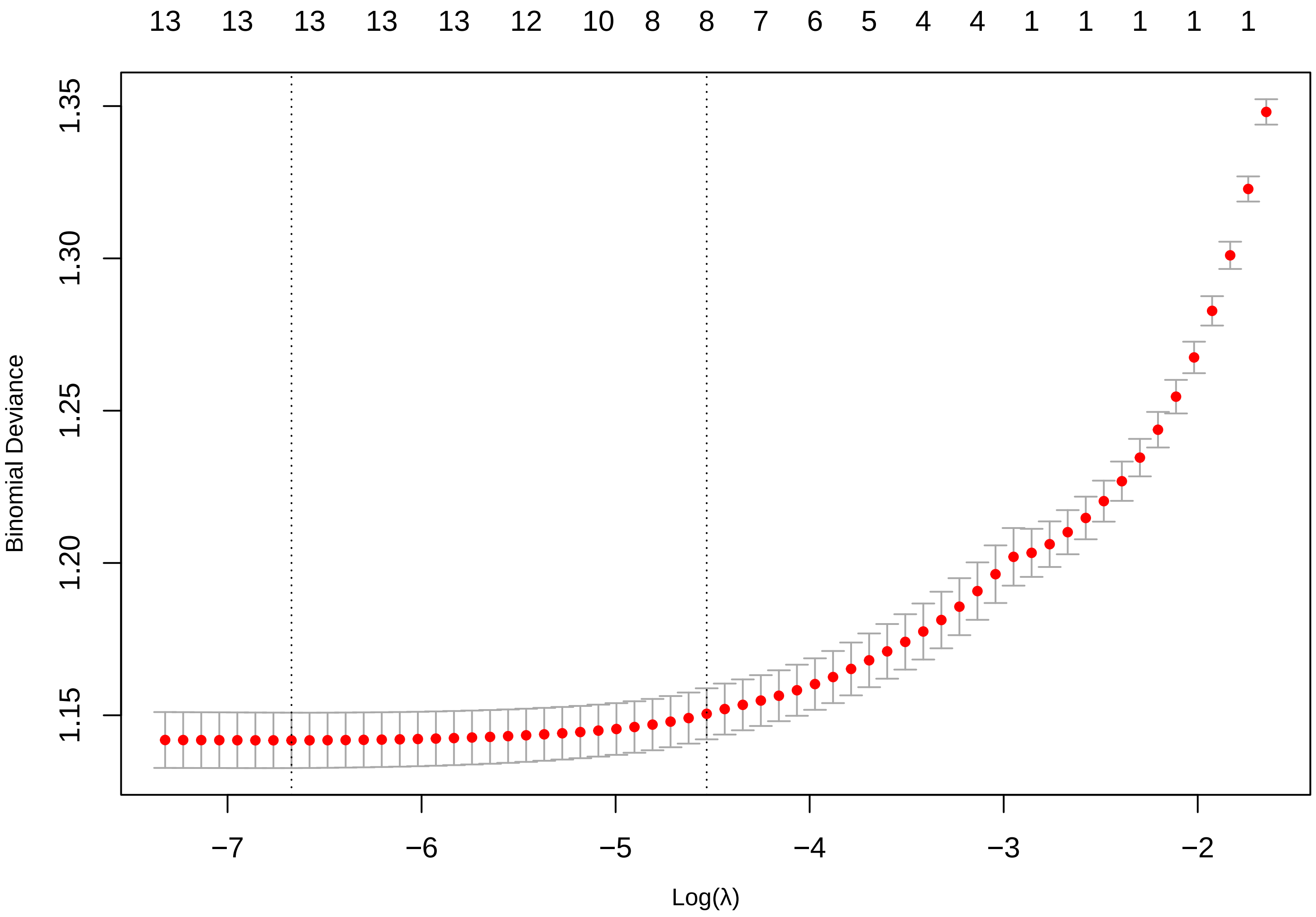

eFigure 4. LASSO coefficient path plot for feature selection in the sarcopenia prediction model.

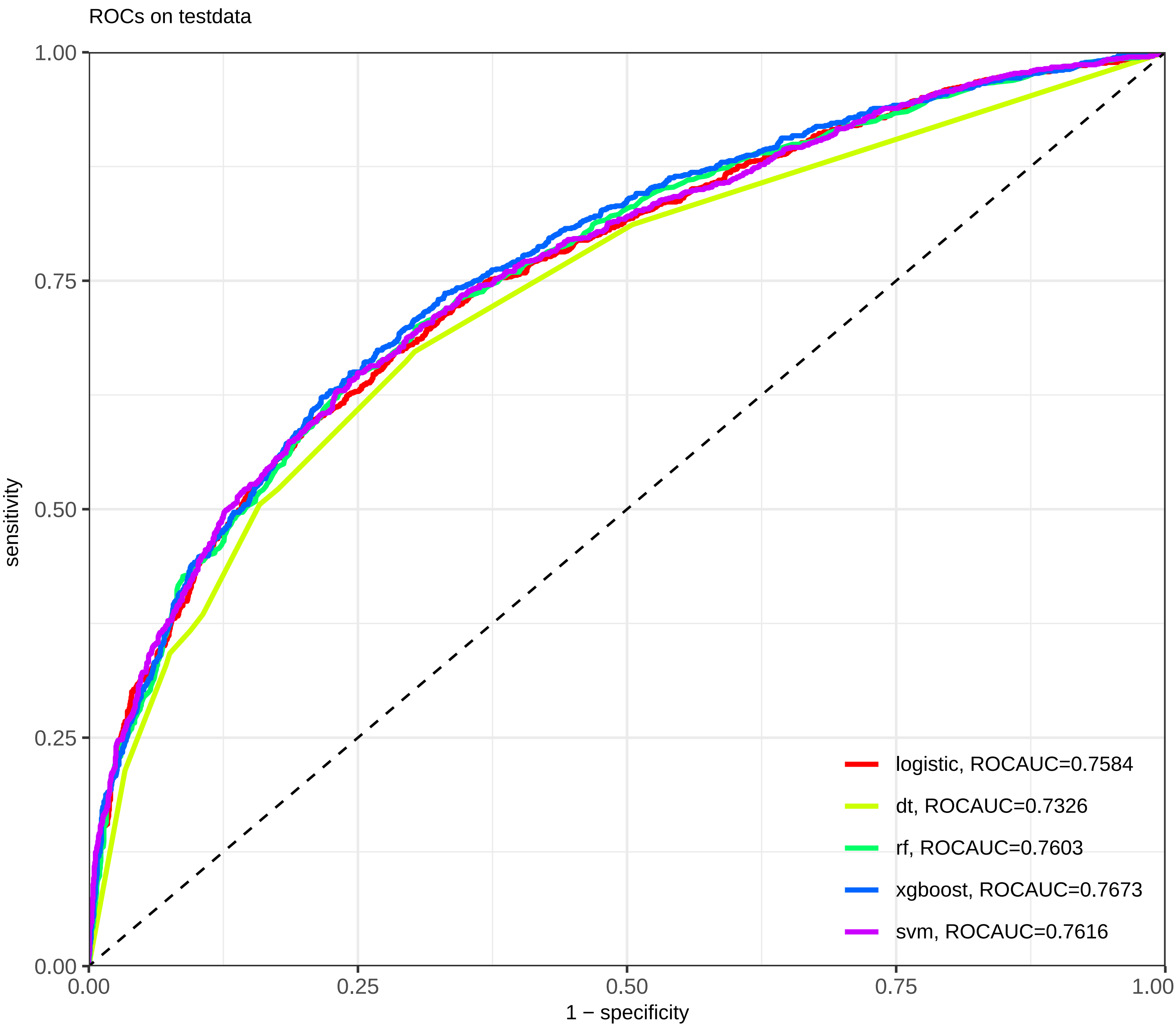

eFigure 5. ROC curves of the five sarcopenia-prediction models on the test set.

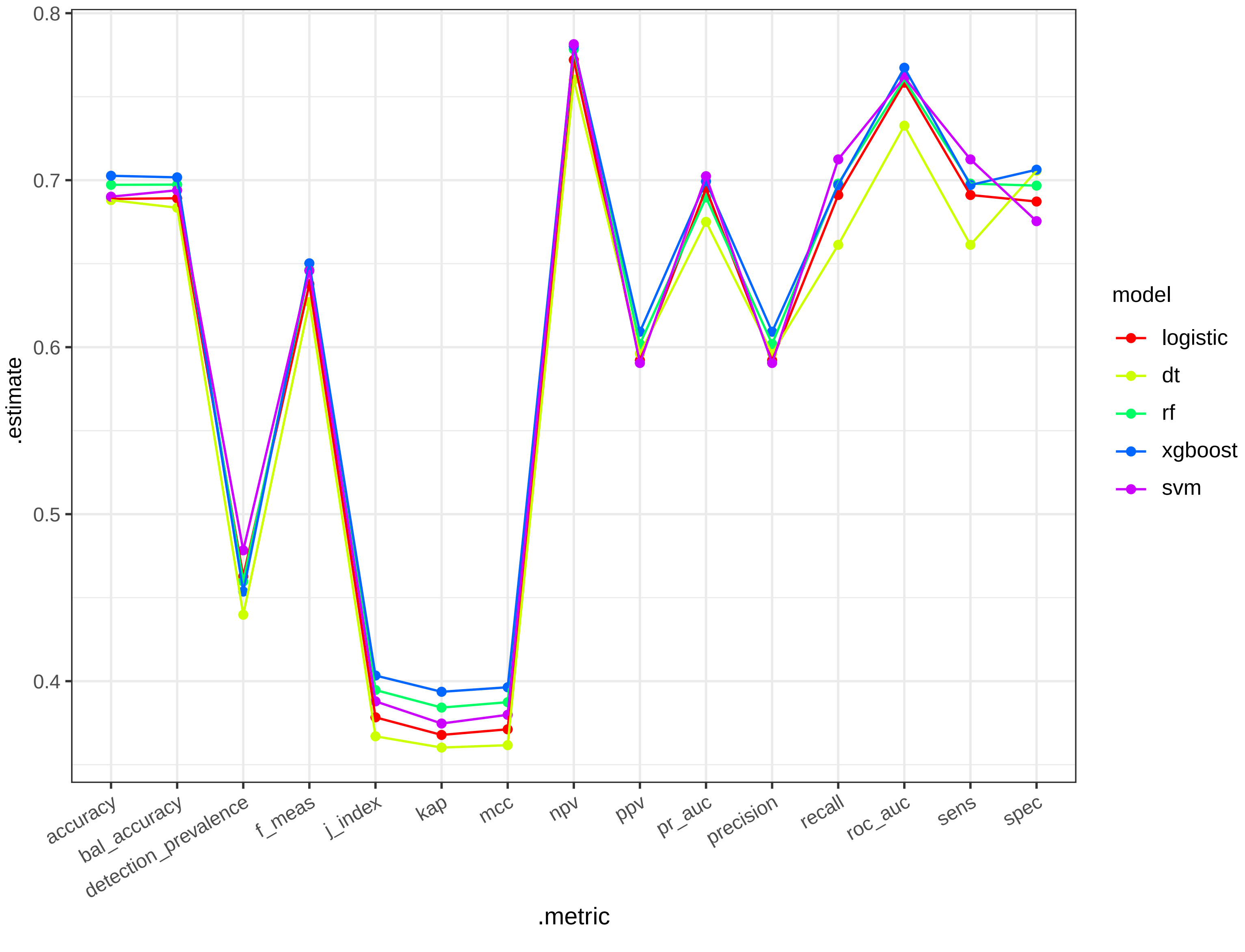

eFigure 6. Parallel-coordinate plot of multi-metric model performance for sarcopenia prediction.

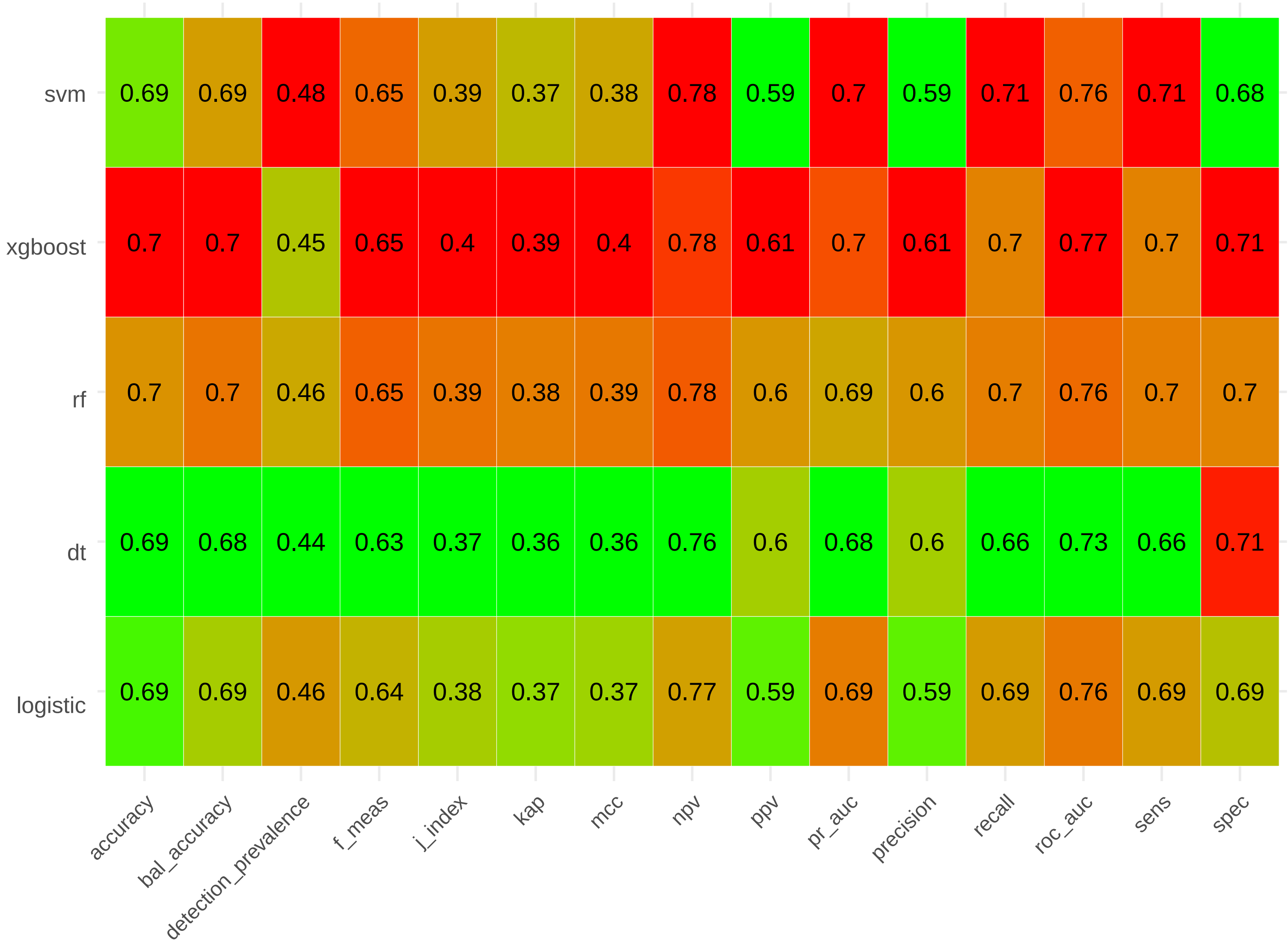

eFigure 7. Heatmap of multi-metric performance comparison for sarcopenia prediction models.

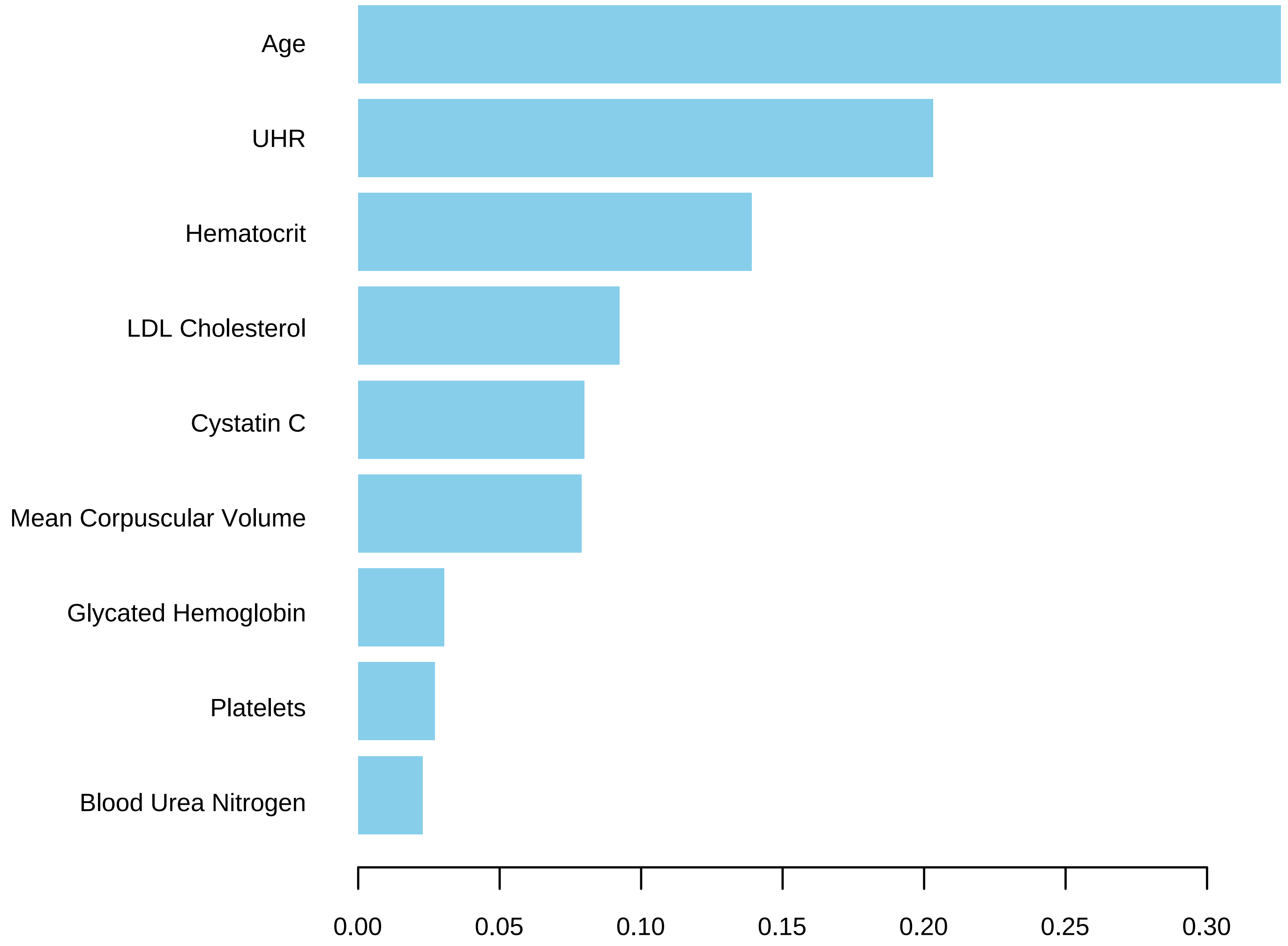

eFigure 8. Variable importance ranking from the XGBoost sarcopenia-prediction model.

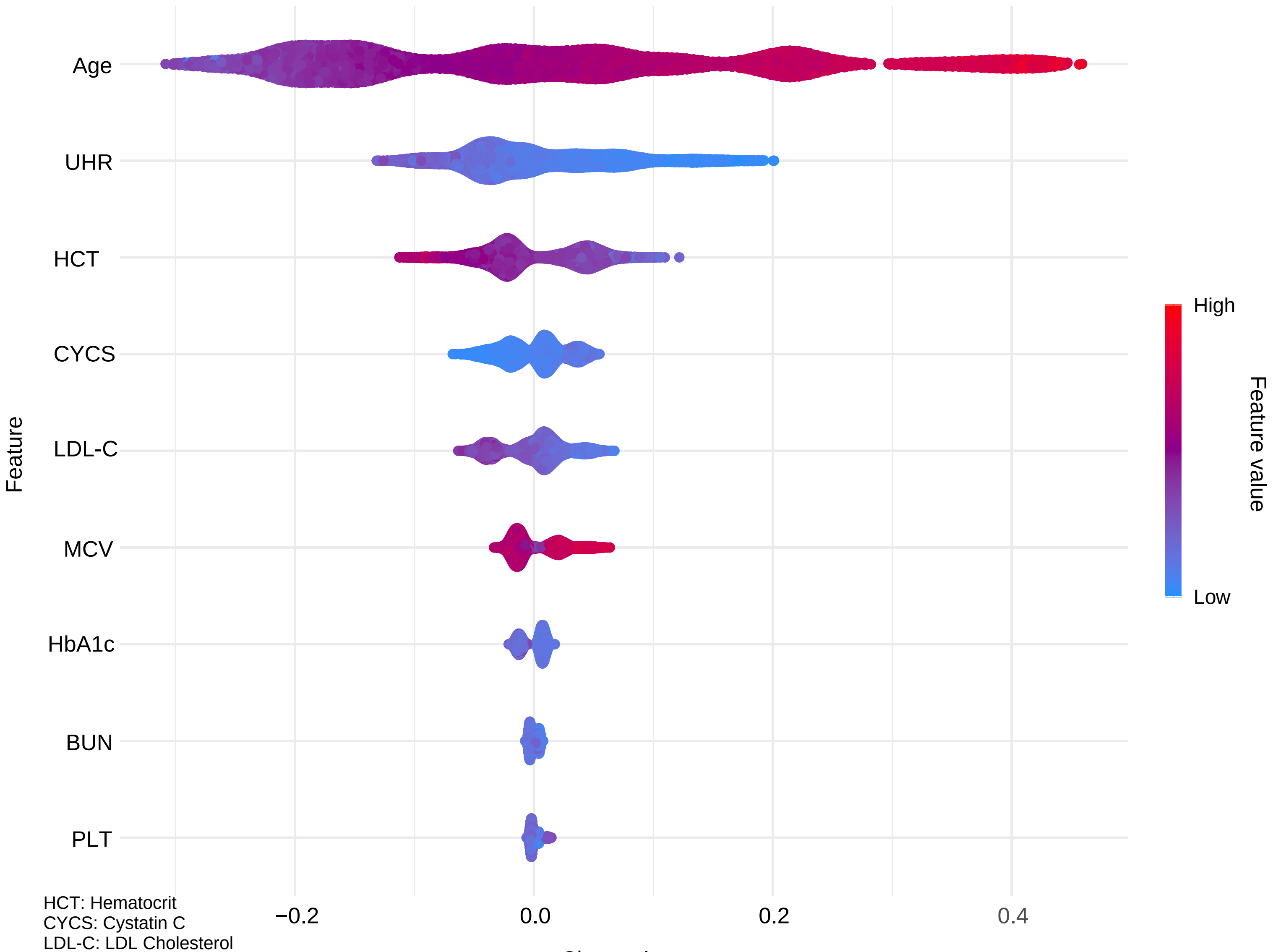

HCT: Hematocrit
CYCS: Cystatin C
LDL-C: LDL Cholesterol
MCV: Mean Corpuscular Volume
HbA1c: Glycated Hemoglobin
BUN: Blood Urea Nitrogen
PLT: Platelets

eFigure 9. SHAP summary plot for the XGBoost sarcopenia-prediction model.

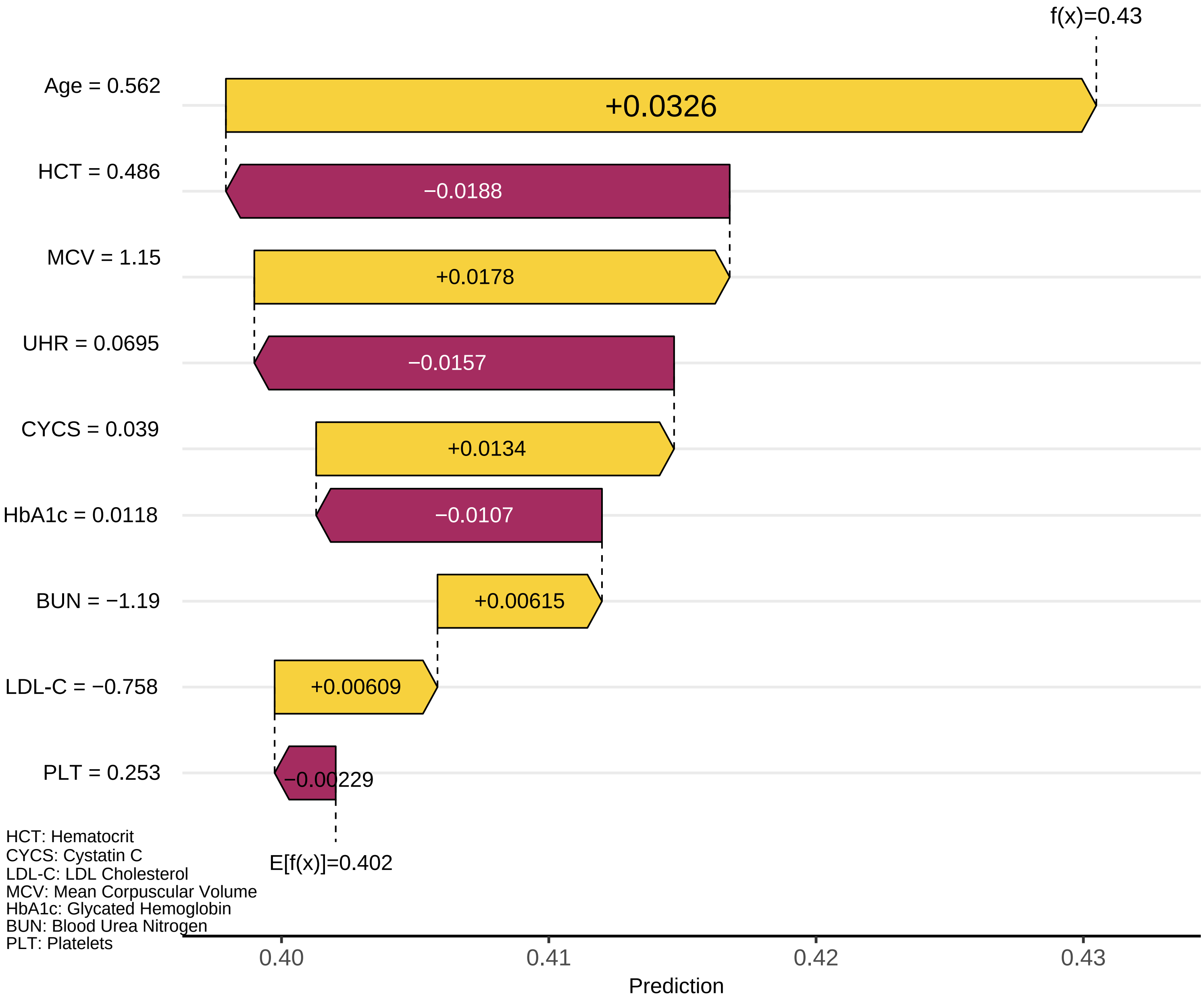

eFigure 10. SHAP force plot illustrating the prediction contribution of each variable in a representative case.

STROBE Statement—checklist of items that should be included in reports of observational studies

	Item No	Recommendation	Page No
**Title and abstract**	1	(*a*) Indicate the study's design with a commonly used term in the title or the abstract	2
		(*b*) Provide in the abstract an informative and balanced summary of what was done and what was found	2-3
**Introduction**			
Background/rationale	2	Explain the scientific background and rationale for the investigation being reported	3-5
Objectives	3	State specific objectives, including any prespecified hypotheses	6
**Methods**			
Study design	4	Present key elements of study design early in the paper	6
Setting	5	Describe the setting, locations, and relevant dates, including periods of recruitment, exposure, follow-up, and data collection	6
Participants	6	(*a*) *Cohort study*—Give the eligibility criteria, and the sources and methods of selection of participants. Describe methods of follow-up<br>*Case-control study*—Give the eligibility criteria, and the sources and methods of case ascertainment and control selection. Give the rationale for the choice of cases and controls<br>*Cross-sectional study*—Give the eligibility criteria, and the sources and methods of selection of participants	6
		(*b*) *Cohort study*—For matched studies, give matching criteria and number of exposed and unexposed<br>*Case-control study*—For matched studies, give matching criteria and the number of controls per case	
Variables	7	Clearly define all outcomes, exposures, predictors, potential confounders, and effect modifiers. Give diagnostic criteria, if applicable	6-8
Data sources/ measurement	8*	For each variable of interest, give sources of data and details of methods of assessment (measurement). Describe comparability of assessment methods if there is more than one group	6-8
Bias	9	Describe any efforts to address potential sources of bias	6,8-9
Study size	10	Explain how the study size was arrived at	6
Quantitative variables	11	Explain how quantitative variables were handled in the analyses. If applicable, describe which groupings were chosen and why	7,9-10
Statistical methods	12	(*a*) Describe all statistical methods, including those used to control for confounding	8-10
		(*b*) Describe any methods used to examine subgroups and interactions	9
		(*c*) Explain how missing data were addressed	6
		(*d*) *Cohort study*—If applicable, explain how loss to follow-up was addressed<br>*Case-control study*—If applicable, explain how matching of cases and controls was addressed<br>*Cross-sectional study*—If applicable, describe analytical methods taking account of sampling strategy	6,8
		(*e*) Describe any sensitivity analyses	10,12-13

Continued on next page

**Results**			
Participants	13*	(a) Report numbers of individuals at each stage of study—eg numbers potentially eligible, examined for eligibility, confirmed eligible, included in the study, completing follow-up, and analysed	6
		(b) Give reasons for non-participation at each stage	6
		(c) Consider use of a flow diagram	6
Descriptive data	14*	(a) Give characteristics of study participants (eg demographic, clinical, social) and information on exposures and potential confounders	11
		(b) Indicate number of participants with missing data for each variable of interest	6
		(c) *Cohort study*—Summarise follow-up time (eg, average and total amount)	
Outcome data	15*	*Cohort study*—Report numbers of outcome events or summary measures over time	
		Case-control study—Report numbers in each exposure category, or summary measures of exposure	
		Cross-sectional study—Report numbers of outcome events or summary measures	11
Main results	16	(*a*) Give unadjusted estimates and, if applicable, confounder-adjusted estimates and their precision (eg, 95% confidence interval). Make clear which confounders were adjusted for and why they were included	11
		(*b*) Report category boundaries when continuous variables were categorized	7
		(*c*) If relevant, consider translating estimates of relative risk into absolute risk for a meaningful time period	
Other analyses	17	Report other analyses done—eg analyses of subgroups and interactions, and sensitivity analyses	11-15
**Discussion**			
Key results	18	Summarise key results with reference to study objectives	15-16
Limitations	19	Discuss limitations of the study, taking into account sources of potential bias or imprecision. Discuss both direction and magnitude of any potential bias	18-19
Interpretation	20	Give a cautious overall interpretation of results considering objectives, limitations, multiplicity of analyses, results from similar studies, and other relevant evidence	19-20
Generalisability	21	Discuss the generalisability (external validity) of the study results	15,19-20
**Other information**			
Funding	22	Give the source of funding and the role of the funders for the present study and, if applicable, for the original study on which the present article is based	21

*Give information separately for cases and controls in case-control studies and, if applicable, for exposed and unexposed groups in cohort and cross-sectional studies.

Note: An Explanation and Elaboration article discusses each checklist item and gives methodological background and published examples of transparent reporting. The STROBE checklist is best used in conjunction with this article (freely available on the Web sites of PLoS Medicine at http://www.plosmedicine.org/, Annals of Internal Medicine at http://www.annals.org/, and Epidemiology at http://www.epidem.com/). Information on the STROBE Initiative is available at www.strobe-statement.org.
