# Supplementary figures and images for: Uric acid to high-density lipoprotein cholesterol ratio as a novel biomarker for sarcopenia: a national study with machine learning insights
Source: J Gerontol A Biol Sci Med Sci. 2026 May 22;81(7):glag132. doi: 10.1093/gerona/glag132 (PMC13264432; doi:10.1093/gerona/glag132)

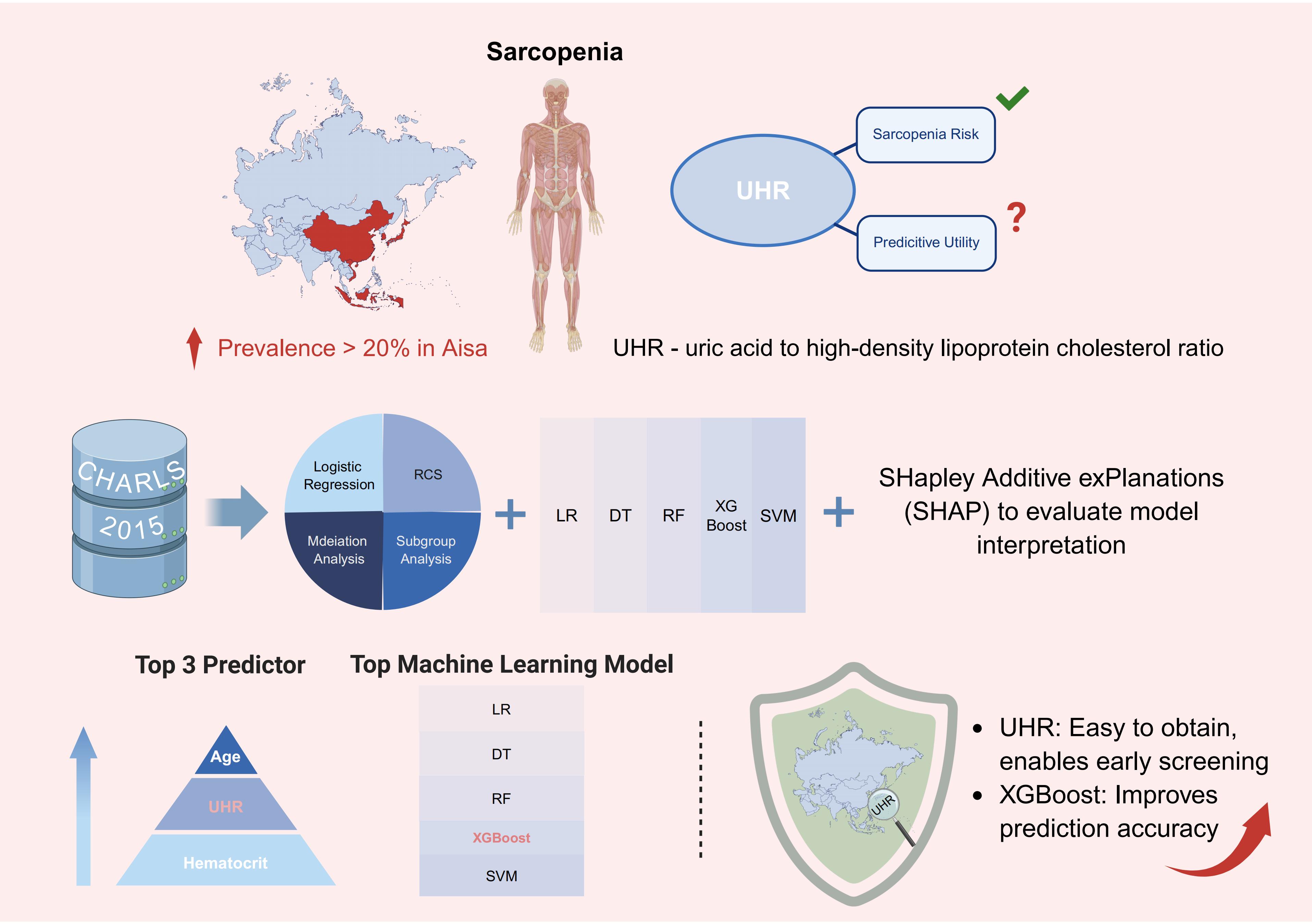

Supplement: glag132_Supplementary_Data [file glag132_supplementary_data.zip › Graphical Abstract.jpg]
